# Next generation sequencing identifies miRNA-based biomarker panel for lupus nephritis

**DOI:** 10.18632/oncotarget.25575

**Published:** 2018-06-15

**Authors:** Yu-Jih Su, I-Chun Lin, Lin Wang, Cheng-Hsien Lu, Yi-Ling Huang, Ho-Chang Kuo

**Affiliations:** ^1^ Department of Rheumatology, Allergy and Immunology, Kaohsiung Chang Gung Memorial Hospital, Chang Gung University College of Medicine, Kaohsiung, Taiwan; ^2^ Department of Pediatrics, Kaohsiung Chang Gung Memorial Hospital, Chang Gung University College of Medicine, Kaohsiung, Taiwan; ^3^ Department of Pediatrics, PoJen Hospital, Kaohsiung, Taiwan; ^4^ Department of Neurology, Kaohsiung Chang Gung Memorial Hospital, Chang Gung University College of Medicine, Kaohsiung, Taiwan; ^5^ Department of Biological Science, National Sun Yat-Sen University, Kaohsiung, Taiwan; ^6^ Department of Neurology, Xiamen Chang Gung Memorial Hospital, Xiamen, Fujian, China; ^7^ Department of Pediatrics and Kawasaki Disease Center, Kaohsiung Chang Gung Memorial Hospital and Chang Gung University College of Medicine, Kaohsiung, Taiwan

**Keywords:** next generation sequencing, lupus, nephritis, microRNA, Immunology

## Abstract

The symptomatology of lupus nephritis (LN) consists of foamy urine and lower leg edema, as well as such systemic manifestations as oral ulcers, arthralgia/arthritis, and lymphadenopathy. However, these symptoms may appear mild and non-specific. If these symptoms are unrecognized, thus delaying treatment, approximately 10% of LN patients will develop permanent kidney damage and end-stage kidney disease. Therefore, the purpose of this study is to identify a surrogate biomarker for the early detection of LN. In this study, we first adopted next generation sequencing (NGS) in order to screen differential expression levels of microRNA between SLE patients with and without LN. The results of both the NGS and the literature review confirmed the potential of 15 microRNAs through real-time qPCR. We further considered clinical laboratory data for additional analysis. In total, 41 microRNAs demonstrated significant differences through NGS screening. We then verified eight microRNAs from NGS and seven microRNAs from the literature review using the real-time qPCR method in peripheral mononuclear cells. Ultimately, mir-125a-5p, miR-146a-5p, and mir-221-3p were found to be statistically significant not only in the screening study but also in the real-time qPCR verification studies. miR-146a-5p was observed to have a significant correlation with clinical biochemistry markers, as well as to be a surrogate biomarker for the early detection of lupus nephritis. This study is the first to show that the intracellular biomarker miR-146a-5p may serve as a useful specific biomarker for the detection of lupus nephritis among lupus patients in the future, regardless of serum albumin levels and spot urine protein/creatinine ratio.

## INTRODUCTION

Systemic lupus erythematosus (SLE) is an autoimmune disease that often affects women of childbearing age and, while symptoms vary, can be fatal if it reaches a major organ system, such as the central nervous system, heart, lungs, or kidneys; this condition is referred to as lupus nephritis (LN). LN presents with such symptoms as foamy urine and lower leg edema, as well as other systemic manifestations like oral ulcers, arthralgia or arthritis, lymphadenopathy, and rashes. However, these symptoms may appear mild and non-specific. If these symptoms go unrecognized and treatment is delayed, nearly 10% of LN patients may develop irreversible kidney damage and end-stage kidney disease; therefore, LN is a major cause of morbidity and even mortality in people with SLE [1, 2]. The successful detection of LN and early treatment can significantly reduce the risk of kidney failure [1]. Therefore, both proper detection and early treatment are vital for effectively managing LN.

Until recently, few cytokine-based biomarker platforms had been developed [3]. Traditionally, diagnosing LN has relied merely on physicians’ judgment based on observing patients’ symptoms, so we decided to find a more convenient and modern method for the early detection and confirmation of LN. Furthermore, many infectious diseases present with symptoms similar to those of LN, thus complicating LN diagnosis and leading to a delay in the optimal and timely treatment of LN. Previous studies have shown that blood microRNAs can function as disease biomarkers [4–6].

In this study, we adopt next generation sequencing (NGS) in order to screen the differential expression levels of microRNA between SLE patients with and without LN, as previously described in our report on Kawasaki Disease [7]. Then, we selected the microRNAs obtained from NGS and those already studied either in animal models or human subjects to further confirm their expression in SLE patients using traditional real-time qPCR to identify the microRNAs that were differentially expressed in SLE patients with and without LN.

## RESULTS

### Comparison between lupus patients with and without nephritis using NGS

The training set consisted of six LC and six LN subjects, whose detailed demographic data is provided in Table 1a. We used these subjects to develop the LN microRNA biomarker panel through NGS. We maintained the quotient between the levels from LN and LC within 0.8 to 1.2, which determined only 41 targeted microRNAs (Table 1b).

**Table 1a:** Demographic data of the patients (six lupus nephritis, six lupus control) in the next generation sequencing study

SLE patients	No lupus nephritis	Lupus nephritis	p-value
Gender (female: male)	6: 0	6: 0	1.000
	mean± standard deviation	mean± standard deviation	
Age (year)	31.5±16.1	38.3±12.3	0.430
Leukocytes (×1000/ml)	7.97±4.24	5.8±1.95	0.298
Hemoglobulin (mg/dL)	12.5±1.76	11.7±1.51	0.497
Neutrophil (%)	84.3±10.9	67.5±12.6	0.062
Lymphocyte (%)	11.9±9.59	26.6±10.7	0.059
Platelets (1000/μL)	228.±35.2	264.±174.	0.696
Erythrocyte Sediment Rate (mm/hr)	35.6±29.5	62±15.0	0.521

**Table 1b:** Comparison between lupus patients with nephritis and without nephritis using next generation sequencing

miR_ID	LN1	LN2	SLE1	SLE2	LN/SLE	SLE/LN
hsa-miR-99b-5p	652.10	495.10	1621.10	1780.10	0.34	2.96
hsa-miR-143-3p	6373.10	3900.10	23219.10	5348.10	0.36	2.78
hsa-miR-486-5p	7143.20	8765.20	24554.20	16698.20	0.39	2.59
hsa-miR-126-5p	2727.10	2501.10	8418.10	3687.10	0.43	2.32
hsa-miR-126-3p	705.10	973.10	2658.10	1107.10	0.45	2.24
hsa-miR-199a-3p	569.20	515.20	1654.20	688.20	0.46	2.16
hsa-miR-340-5p	824.10	563.10	1671.10	785.10	0.56	1.77
hsa-miR-151a-3p	3578.10	3057.10	8024.10	3571.10	0.57	1.75
hsa-miR-151a-5p	2621.10	2187.10	4969.10	3185.10	0.59	1.70
hsa-miR-223-3p	10565.10	4595.10	18045.10	6138.10	0.63	1.60
hsa-miR-125a-5p	2011.10	1974.10	3209.10	2987.10	0.64	1.55
hsa-miR-26b-5p	17699.10	24701.10	34869.10	30014.10	0.65	1.53
hsa-miR-148b-3p	2051.10	1635.10	3218.10	2210.10	0.68	1.47
hsa-miR-10a-5p	3252.10	3187.10	4805.10	4549.10	0.69	1.45
hsa-miR-98-5p	1760.10	1471.10	2379.10	2274.10	0.69	1.44
hsa-miR-22-3p	13949.10	12183.10	16374.10	20259.10	0.71	1.40
hsa-miR-27b-3p	6355.10	4758.10	8060.10	7420.10	0.72	1.39
hsa-miR-146a-5p	9760.10	16025.10	18802.10	16776.10	0.72	1.38
hsa-miR-103a-3p	6925.20	6467.20	10036.20	8309.20	0.73	1.37
hsa-miR-191-5p	61317.10	32521.10	57626.10	65608.10	0.76	1.31
hsa-miR-148a-3p	6827.10	5994.10	11598.10	4849.10	0.78	1.28
hsa-let-7f-5p	23045.20	25408.20	34193.20	27428.20	0.79	1.27
hsa-miR-24-3p	1160.20	958.20	1328.20	1338.20	0.79	1.26
hsa-miR-222-3p	950.10	691.10	1277.10	754.10	0.81	1.24
hsa-miR-221-3p	2210.10	2968.10	3945.10	2357.10	0.82	1.22
hsa-let-7a-5p	22316.30	22811.30	26797.30	27846.30	0.83	1.21
hsa-miR-181a-5p	132236.20	142144.20	103236.20	119400.20	1.23	0.81
hsa-miR-142-5p	89281.10	94036.10	66802.10	80091.10	1.25	0.80
hsa-miR-28-3p	7173.10	5093.10	4937.10	4879.10	1.25	0.80
hsa-miR-29a-3p	10099.10	12856.10	9120.10	9168.10	1.26	0.80
hsa-miR-342-3p	5349.10	6968.10	4102.10	5678.10	1.26	0.79
hsa-miR-29c-3p	1412.10	1720.10	1196.10	1233.10	1.29	0.78
hsa-miR-181a-3p	1107.10	730.10	443.10	967.10	1.30	0.77
hsa-miR-146b-5p	47962.10	65169.10	37013.10	47586.10	1.34	0.75
hsa-miR-423-5p	1735.10	1128.10	990.10	1127.10	1.35	0.74
hsa-miR-484	2637.10	1751.10	1133.10	2038.10	1.38	0.72
hsa-miR-197-3p	1437.10	637.10	681.10	797.10	1.40	0.71
hsa-miR-181b-5p	4586.20	4460.20	2920.20	3225.20	1.47	0.68
hsa-miR-363-3p	2164.10	1365.10	1171.10	1003.10	1.62	0.62
hsa-miR-150-5p	83540.10	96534.10	40874.10	63727.10	1.72	0.58
hsa-miR-146b-3p	1280.10	1340.10	756.10	728.10	1.77	0.57

Abbreviations: hsa-miR, human microRNA; LN1, first three lupus nephritis patients’ microRNA mixture; LN2, second three lupus nephritis patients’ microRNA mixture; SLE, systemic lupus erythematosus; SLE1, first three non-lupus nephritis SLE patients’ microRNA mixture; SLE2, second three non-lupus nephritis SLE patients’ microRNA mixture.

### Demographic data of the study subjects

In total, we enrolled 78 lupus patients, including 56 lupus control (LC, denoting non-LN SLE patients) and 22 LN subjects (LN, lupus nephritis patients) in this study. These patients made up the confirmation test set, which was utilized to evaluate the performance of the LN microRNA biomarker panel developing through the previously mentioned NGS screening set. Table 2 provides the demographic data of all the SLE subjects. The leukocyte count and the neutrophil percentage of LN patients are significantly higher than those of LC patients (both *p* < 0.05), while the lymphocyte percentage is significantly lower in LN than in LC patients (*p* < 0.05). The biochemistry of serum albumin, creatinine, and urine protein: creatinine ratio differed significantly between the LN and LC groups (all *p* < 0.05).

**Table 2:** Demographic data of subjects in the confirmation set in this study

SLE patients	No lupus nephritis(α)	Lupus nephritis(β)	p-value
Gender (female: male)	53:3	17:5	0.03*
	mean± standard deviation (*n* = 56)	mean± standard deviation (*n* = 22)	
Age (year)	28.72±14.87	28.3±14.79	0.91
Leukocytes (×1000/ml)	5.73±2.92	7.53±3.57	0.03*
Hemoglobulin (mg/dL)	12.04±1.56	11.60±2.46	0.45
Neutrophil (%)	62.49±11.95	73.07±12.68	0.002*
Lymphocyte (%)	29.36±11.00	19.77±10.67	0.002*
Platelets (1000/μL)	254.47±124.81	226.24±70.82	0.34
Alanine aminotransferase (U/dL)	16.88±12.84	25±0.21	0.21
Erythrocyte Sediment Rate (mm/hr)	28.84±29.15	27.07±23.66	0.84
Albumin (mg/dL)	4.25±0.42	3.27±0.89	0.01*
anti-dsDNA (U/dL)	255.58±195.41	307.99±156.27	0.40
C3 (mg/dL)	85.69±25.70	75.95±23.61	0.16
C4 (mg/dL)	18.62±18.28	13.44±6.05	0.24
Creatinine (mg/dL)	0.63±0.12	1.35±1.26	0.03*
Urine protein/Creatinine	168.58±149.48	1568.89±1468.85	0.003*

Abbreviations: SLE, systemic lupus erythematosus;

§ Data presented with mean ± SD (Standard deviation)

Continuous variables between two groups were compared using Student's T-test, between α and β. α: SLE with no lupus nephritis, β: SLE with lupus nephritis.

*: indicates *p*-value<0.05,

Gender was compared using Fisher's Exact Test.

### Real-time qPCR validation of microRNA expression in peripheral mononuclear cells

We selected 15 microRNA targets and tested them using real-time qPCR validation for microRNA expression in peripheral mononuclear cells, as shown in Table 3. mir-125a-5p, miR-146a-5p, and mir-221-3p each showed a significant correlation between the NGS screening study and the real-time qPCR verification studies, and all three of them differed significantly between the LN and LC patient groups (all *p* < 0.05). Only one of the microRNAs that we selected from our literature review, miR-193b, demonstrated a significant differed between the LN and LC groups (*p* < 0.05), but said difference did not reach significance through the NGS screening study done at the beginning of this study (Table 1b).

**Table 3:** Confirmation of selected microRNA levels in peripheral mononuclear cells and between lupus patients with (β) and without (α) nephritis

SLE patients	No lupus nephritis(α)	Lupus nephritis(β)	p-value
Gender (female: male)	53:3	17:5	0.03*
	mean± standard deviation (*n* = 56)	mean± standard deviation (*n* = 22)	
**Mononuclear cell microRNA ΔCT and normalized with U6 as a control**			
miR-29b-3p^##^	1.01±0.54	1±1.09	0.979
**miR-125a-5p**^#^	1.54±1.68	1±0.70	0.048*
miR-125a-3p^##^	1.22±0.99	1±0.84	0.335
**miR-146a-5p**^#^	1.47±1.12	1±0.71	0.032*
miR-155^##^	1.07±0.57	1±0.62	0.628
miR-193b^##^	1.71±1.42	1±0.91	0.013*
miR-574-3p^##^	1.03±0.62	1±0.34	0.800
**miR-221-3p**^#^	1.77±0.26	1±0.13	0.011*
miR-21-5p^##^	0.84±0.50	1±0.17	0.383
miR-23a-3p^##^	1.09±0.10	1±0.16	0.647
let-7a-5p^#^	1.20±0.75	1±0.34	0.185
miR-197-3p^#^	1.03±0.71	1±0.34	0.849
miR-181a-5p^##^	1.14±0.53	1±0.43	0.248
miR-150-5p^#^	1.26±0.71	1±0.75	0.270
miR-223-3p^##^	0.95±0.96	1±0.57	0.764

Abbreviations: SLE, systemic lupus erythematosus;

§ Data presented with mean ± SD (Standard deviation)

Continuous variables between two groups were compared using Student's T-test, between α and β. α: SLE with no lupus nephritis, β: SLE with lupus nephritis.

*: indicates *p*-value < 0.05,

Gender was compared using Fisher's Exact Test.

### Correlation between clinical markers and the three identified microRNAs

Clinical leukocyte, neutrophil, lymphocyte, and creatinine levels significantly correlated with mir-146a-5p (all *p* < 0.05, Table 4). These four clinical markers were derived from the six markers that significantly differed between lupus patients with and without lupus nephritis (all *p* < 0.05, Table 2). The albumin level and the urine creatinine/protein ratio level did not correlate with mir-146a-5p (Table 4), indicating that mir-146a-5p may function as a marker even before the albumin and urine creatinine/protein ratio level change (Table 2 and Table 4).

**Table 4:** Correlation between clinical laboratory data and the three microRNAs screened using next generation sequencing and confirmed with individual real-time PCR

microRNAs selected from the next generation sequencing study		miR-125a-5p	miR-146a-5p	miR-221-3p
Clinical markers:				
Leukocyte and differential counts				
Leukocytes	γ	-.08	.24*	.10
	p	.51	.05*	.50
Neutrophil	γ	-.09	.33*	.06
	p	.49	.01*	.69
Lymphocyte	γ	.08	-.35*	-.03
	p	.51	.01*	.84
Biochemistry tests				
Albumin	γ	.01	-.15	-.10
	p	.97	.46	.66
Creatinine	γ	.12	.36*	.19
	p	.36	.01*	.24
Urine protein loss				
Urine protein/Creatinine	γ	.19	.30	.27
	p	.33	.12	.28

γ:Pearson correlation; p: *p*-value, significant if *p* < 0.05.

*: indicates *p* < 0.05

### Preliminary longitudinal follow-up study in LC and LN patients

All three of the selected microRNAs are significantly elevated after LC patients receive treatment (all *p* < 0.05). In the follow-up study, mir-125a-5p was significantly elevated in LN patients after treatment (*p* < 0.05). Meanwhile, mir-146a-5p was elevated but, although very close, did not reach statistical significance (*n* = 8, *p* = 0.053) (Supplementary Table 1). The microRNAs did not reach statistical significance between the LC and LN subgroups in the preliminary follow-up study (Table 1).

## DISCUSSION

Diagnosing LN currently depends on a physician's experience, so accurately doing so can be difficult. According to our literature review, this study is the first in which NGS, qPCR, and a preliminary longitudinal follow-up study were used to identify a highly accurate microRNA-based biomarker panel for LN. We extracted RNA samples from white blood cells and created two pooled LC and two pooled LN RNA libraries that were sequenced using the Illumina NGS platform, followed by miRSeq [8] analysis to determine the microRNA expression profiles (Table 2). In order to assess whether the LC and LN samples could be differentiated using microRNA expression profiles, we performed clustering analysis. More than a dozen microRNAs were capable of distinguishing LC from LN samples and thus could potentially serve as biomarkers for lupus nephritis (Table 2).

To further validate the overall patient pool of 56 LC and 22 LN subjects using qPCR, we selected 15 microRNAs with a NGS abundance greater than 1000 TPM (transcripts per million) in at least one library, as well as an average fold change greater than 1.2 between LC and LN libraries, and whose microRNA was indicated through the regulation mononuclear cellular function. We then observed three microRNAs whose qPCR expression preferences agreed with the ones we found using NGS (Table 3).

As women have a higher LN incidence rate than men do, we also examined whether the 10 microRNAs were differentially expressed to a level of significance between male and female subjects and thus may have created a bias when serving as a disease biomarker. While the gender difference between LN and non-LN was statistically different (*p* = 0.03), each individual microRNA did not differ significantly between different patient genders. We compared these microRNAs between male and female patients and found that only miR-146a-5p, which correlated with clinical markers, was not gender dependent, while the remaining intracellular biomarker microRNAs were not gender specific.

Lastly, we correlated the clinical markers with these confirmed microRNA levels and found that clinical leukocyte, neutrophil, lymphocyte, and creatinine levels demonstrated significant correlations with mir-146a-5p (all *p* < 0.05, Table 4). The positive correlation between mir-146a-5p and leukocyte, neutrophil, and creatinine levels, as well as the negative correlation of the lymphocyte percentage with mir-146a-5p, differentially demonstrated the natural characteristic clinical presentation between LN and LC, as shown in Table 2. However, mir-146a-5 is not correlated with the albumin level or the urine protein/creatinine level (both *p* > 0.05). This finding supports our hypothesis that using mir-146a-5p in clinical situations as a marker to detect LN even before the albumin level decreases or the urine protein/creatinine level increases.

To provide better insight into these three specific microRNAs, we compared microRNAs before and after treatment in 17 random patients (Supplementary Table 1). The 17 patients included nine LC patients and eight LN patients, and the supplementary follow-up study indicated that the three microRNAs, mir-146a-5p, mir-125a-5p, and mir-221-3p, respond to clinical conditions to a significant extent in LC patients, which may be related to either SLE disease activity or SLE organ specificity, such as subclinical lupus nephritis. All three microRNAs are significantly elevated after treatment in LC (all *p* < 0.05). In the follow-up study, the mir-125a-5p was significantly elevated in LN patients following treatment (*p* < 0.05). Meanwhile, mir-146a-5p was elevated but did not reach statistical significance (*n* = 8, *p* = 0.053). The elevation trend of mir-125a-5p and mir-146a-5p in LN confirmed our belief that the three microRNAs are organ specific, i.e. nephritis, both in our NGS and in our intracellular microRNA real-time qPCR studies.

miR-146a-5p has been reported to be dysregulated in type I diabetes mellitus patients and may be involved in the pathways related to immune function, cell survival, proliferation, and insulin biosynthesis and secretion [9]. Lo *et al.* [10] reported that miR-146a-5p played a role in endothelial inflammation by modulating interleukin-1 receptor-associated kinase-1 (IRAK-1), thus suggesting that miR-146a-5p may be an effective treatment target for diabetic vasculopathy. One study recently reported that the suppression of IRAK1 or IRAK4, but not type I interferon signaling, can prevent LN in mice, which indicated the importance of miR-146a-5p and IRAK1 in the pathogenesis of LN [11]. Furthermore, Wang-Renault *et al.* [12] demonstrated that miR-146a-5p was deregulated in T cells from patients diagnosed with primary Sjögren's syndrome.

In this study, we checked several target mRNA levels that were assumed to be suppressed by mir-125a-5p, miR-146a-5p, and mir-221-3p, including SMAD4, ZNF652, EIF5A2, TRAF6, ETS1, and IRAK1, using real-time PCR in a cross-sectional fashion. In the preliminary follow-up study, the mRNAs were tested to be higher in LN from gene ZNF652 and gene ETS1 (both *p* < 0.05) and borderline significant from gene TRAF6 (*p* = 0.05) and gene IRAK1 (*p* = 0.08) (data not shown). As a result, the three microRNAs that we found in this study were assumed to be functionally active.

To the best of our knowledge, several research articles have previously demonstrated that serum-free microRNA or intracellular microRNA correlated with lupus nephritis. Our report further demonstrated an early correlation between mir-146a-5p and leukocyte counts and kidney creatinine levels that were only minimally elevated over 1.35±1.26 mg/dL. Prior to noting significant proteinuria, the albumin and urine protein/creatinine levels were still within normal limits, which were 3.27±0.89mg/dL and 1568.89±1468.85, respectively, and the three microRNAs were capable of representing LN. Furthermore, our follow-up study demonstrated that mir-125a-5p is LN specific and elevated after LN patients receive treatment. In fact, many mRNAs were detected to be significantly differentially expressed between LN and LC patients, which may be attributed to the three microRNAs we selected in this study. In the future, this intracellular biomarker can be useful for the early detection of LN in lupus patients.

## MATERIALS AND METHODS

### Study patients

We enrolled 22 LN and 56 lupus control (LC) subjects from Kaohsiung Chang Gung Memorial Hospital in Taiwan in this study and used ‘LC’ to represent lupus control subjects, which included patients with lupus but that had not been diagnosed with lupus nephritis by a physician. Meanwhile, ‘LN’ indicates patients with lupus nephritis, including patients with lupus and that have been diagnosed as having nephritis caused by lupus by a physician according to previous publications [13, 14]. Table 1 shows the detailed information of the subjects, including age, gender, clinical symptoms, and laboratory data. Upon receiving approval from Chang Gung Memorial Hospital's Institutional Review Board (IRB No. 103-7505B, 104-7089C, 105-4874C, and 1612150063), we collected whole blood samples from the subjects. We then prospectively evaluated patients with a definitive diagnosis of SLE who were followed up at the Rheumatology Out-patient Clinic for more than six months. The diagnostic criteria for SLE were based on the 1997 revision of the 1982 American College of Rheumatology (ACR) classification criteria for SLE [15], while the clinical assessment of SLE disease activity was based on the SLE disease activity index (SLEDAI) [16].

This study had two enrollment parts. The first part involved the six LC and six LN subjects that comprised the screening set and were utilized to develop potential microRNA biomarker panel targets that could differentiate LC from LN through next generation sequencing (NGS). All twelve of these SLE patients were taking steroids and immune-modifying medications that were not altered during the entire study period. For comparison, the six lupus controls were age- and gender-matched with the other six lupus nephritis patients. After completing the NGS screening set, we eliminated the microRNAs whose LN and LC quotient levels were within 0.8 to 1.2. Therefore, we were able to keep only the potential microRNA panel targets that were significantly different between LN and LC.

After comparing LC and LN and establishing the potential microRNA panel targets, we recruited additional SLE patients for the second confirmation part, which included a total of 56 LC and 22 LN subjects. In addition to the microRNA data from the NGS set, we performed a literature review and selected various microRNA targets of interest to test in this confirmation set. In this part, we tested the selected microRNA targets in each lupus patient to determine any specific target microRNA that could distinguish between LN patients and LC patients. The Institutional Review Committee on Human Research approved the study protocol, and we obtained the informed consent from all the participants. Any patients with autoimmune diseases other than SLE or fever or other infectious disorders that may affect the kidney were excluded.

### Clinical assessments

All 78 subjects underwent complete medical examinations upon being enrolled. Complement levels and anti-double strand DNA levels were collected upon enrollment and then taken regularly. We also collected various clinical biomarkers, such as demographic data, biochemistry data, urine protein levels, and erythrocyte sediment rate, for further analysis.

### Sample collection

We immediately separated the whole blood into plasma and blood cells (i.e., leukocytes and erythrocytes) using centrifugation at 2,500 rpm (150 ×g) for 20 minutes. Leukocytes were separated from erythrocytes by 4.5% dextran sedimentation at a ratio of 1:5 to separate the leukocytes from the red blood cells (RBCs). We then separated the leukocytes into polymorphonuclear cells (PMNs) and mononuclear cells (MNCs) using density gradient centrifugation in Ficoll-Paque (Amersham Pharmacia Biotech) at a ratio of 2:1 at 1500 rpm for 30 minutes, as described in our previous articles, so that the two layers would have no significant mixing [17, 18]. We further processed the MNC collections with the Direct-zol™ RNA MiniPrep kit (ZYMO RESEARCH) to extract total RNAs, followed by NGS and/or qPCR assays.

### microRNA profiling with NGS

The RNA samples from the MNC collections of the six LN and six LC subjects, which were gender- and age-matched, were evenly pooled with three LN vs. three LC to generate two LC- versus LN-pooled RNA libraries. We prepared the pooled libraries following the TruSeq^®^ Small RNA (Illumina) sample preparation protocol and sequencing using the Illumina MiSeq platform. We used miRSeq to analyze the generated raw data, evaluate the overall sequencing qualities and determine the microRNA expression profiles, as described in our previous report on Kawasaki disease [7].

### Confirming selected microRNA Levels

#### Real-time qPCR validation of microRNA expression abundances

We used the TaqMan™ MicroRNA Reverse Transcription Kit (Applied Biosystems) to prepare the cDNA. Each reaction required 20 ng of total RNA from peripheral blood mononuclear cells (PBMCs). We carried out reverse-transcription reactions using a Veriti 96-well thermal cycler (Applied Biosystems) following the manufacturer's instructions. We adopted a reverse-transcription product to carry out the qPCR reaction and performed quantitative RT-PCR using the 7500 Real-Time PCR System (Applied Biosystems) and the TaqMan Universal PCR Master Mix II without UNG (Applied Biosystems). The real-time PCR cycling conditions were 95°C for 10 min, followed by 40 cycles of 95°C for 15 s and 60°C for 1 min. We determined microRNA expression abundances using ΔCt values, with the small nucleolar RNA U6 as the endogenous control. We previously measured the Ct values of U6, RNU24, RNU44, and RNU48 in samples and observed that their standard deviations were 0.77, 1.05, 1.21, and 0.81, respectively. Therefore, in the current study, U6 is the least variable internal control gene that we measured.

### Statistical analysis

All data in this study were expressed as mean ± SD or median (inter-quartile range). Categorical variables were compared using the Chi-square test or Fisher's exact test, as necessary. Continuous variables were compared between the two groups using Student's t-test. A variable was considered statistically significant if *p* < 0.05. All statistical calculations were performed using the SAS software package, version 9.1 (2002, SAS Statistical Institute, North Carolina).

## CONCLUSIONS

In this study, we found four intracellular microRNAs: miR-146a-5p, miR-193b, miR-125a-5p, and miR-221-3p, within the peripheral mononuclear cells that were capable of differentiating between lupus patients with nephritis and those without nephritis. miR-146a-5p has been associated with peripheral blood cell percentage and the creatinine levels in lupus nephritis patients. In the preliminary follow-up study, mir-125a-5p was significantly elevated in LN patients following treatment (*p* < 0.05). mir-146a-5p was also elevated but without statistical significance (*p* = 0.053). Several mRNAs were selected for testing, which mRNAs from gene ZNF652 and gene ETS1 expressed with significant difference between LN and LC (both *p* < 0.05) and borderline significance from gene TRAF6 (*p* = 0.05) and gene IRAK1 (*p* = 0.08).

## SUPPLEMENTARY MATERIALS TABLE


