# Supplementary material for: Next generation sequencing identifies miRNA-based biomarker panel for lupus nephritis
Source: Oncotarget. 2018 Jun 15;9(46):27911–9. doi: 10.18632/oncotarget.25575 (PMC6021342; doi:10.18632/oncotarget.25575)
Supplement: Supplementary file 1 [file oncotarget-09-27911-s001.pdf]

Next generation sequencing identifies miRNA-based biomarker panel for lupus nephritis

SUPPLEMENTARY MATERIALS

Supplementary Table 1: Serial follow up of intracellular microRNA levels in nine lupus control and nine lupus nephritis patients

Serial follow up	Before treatment	Post treatment	
Intracellular microRNA	mean± standard deviation		*p* value
Lupus controls ($n = 9$)			
miR-146a-5p	0.477±0.345	1±0.300	0.001*
miR-125a-5p	0.023±0.019	1±0.923	0.013*
miR-221-3p	0.362±0.385	1±0.855	0.023*
Lupus nephritis ($n = 8$)			
miR-146a-5p	0.482±0.417	1±0.757	0.053
miR-125a-5p	0.114±0.253	1±0.871	0.036*
miR-221-3p	0.381±0.264	1±0.951	0.14

*, indicates $p < 0.05$
